# Mental health literacy in an educational elite – an online survey among university students

**DOI:** 10.1186/1471-2458-5-44

**Published:** 2005-05-09

**Authors:** Christoph Lauber, Vladeta Ajdacic-Gross, Nadja Fritschi, Niklaus Stulz, Wulf Rössler

**Affiliations:** 1 Psychiatric University Hospital, Zurich, Switzerland

## Abstract

**Background:**

Mental health literacy is a prerequisite for early recognition and intervention in mental disorders. The aims of this paper are to determine whether a sample of university students recognise different symptoms of depression and schizophrenia and to reveal factors influencing correct recognition.

**Methods:**

Bivariate and correspondence analyses of the results from an online survey among university students (n = 225).

**Results:**

Most participants recognised the specific symptoms of depression. The symptoms of schizophrenia were acknowledged to a lower extent. Delusions of control and hallucinations of taste were not identified as symptoms of schizophrenia. Repeated revival of a trauma for depression and split personality for schizophrenia were frequently mistaken as symptoms of the respective disorders. Bivariate analyses demonstrated that previous interest in and a side job related to mental disorders, as well as previous personal treatment experience had a positive influence on symptom recognition. The correspondence analysis showed that male students of natural science, economics and philosophy are illiterate in recognising the symptoms depression and schizophrenia.

**Conclusion:**

Among the educational elite, a wide variability in mental health literacy was found. Therefore, it's important for public mental health interventions to focus on the different recognition rates in depression and schizophrenia. Possibilities for contact must be arranged according to interest and activity (e.g., at work). In order to improve mental health literacy, finally, education and/or internship should be integrated in high school or apprenticeship curricula. Special emphasis must be given towards the effects of gender and stereotypes held about mental illnesses.

## Background

Mental health literacy is defined as the ability to gain access to, understand and use information in ways that promote and maintain good mental health. It refers to knowledge and beliefs about mental disorders that aid their recognition, management or prevention. It also includes the ability to recognise specific disorders, know risk factors and causes, know self-treatments and available professional help, and it is an attitude that promotes recognition and appropriate help-seeking [1]. The concept of mental health literacy implies that it is crucial to increase the *public's *knowledge about mental health and mental diseases since it is a *prerequisite *for early recognition and intervention in mental disorders.

The lifetime risk of developing any mental disorder is nearly 50% [2]. Thus, during a lifetime almost everybody has direct contact with an affected person. Recognition of mental disorders is essential as it influences a person's *attitude and behaviour *towards those affected [3]. Moreover, mental health literacy is an important determinant of *help-seeking behaviour*. Less knowledge about mental illness and its symptoms and possible treatment approaches are negatively associated with health care use [4]. In addition, mental disorders account for a considerable *economic and emotional burden*. Five out of ten leading illnesses associated with disease burden are psychiatric disorders. WHO estimates that in 2020 major depression will become the second most leading cause of disease burden at all [5]. Mental disorders are generally life shortening [6]. As a consequence, mental health literacy has gained increased attention within the last few years [7-20].

Recent studies have shown that mental health literacy is low, regardless of the population considered [21-25]. Although people distinguish abnormal from normal behaviour at a relatively satisfactory level, the recognition of a particular diagnosis is poor. However, in everyday life, people complain about *specific symptoms*. So far, studies have assessed mental health literacy exclusively by means of vignettes, i.e. short stories depicting a person with clinical symptoms of a particular diagnosis, mostly depression or schizophrenia [10,24,26-28]. Although this method allows one to draw conclusions about the recognition of a disorder in general, no statement about the recognition of the specific symptoms is possible. Thus, we investigated in a sample of university students the recognition of different symptoms of depression and schizophrenia. The following questions were used to assess the students' recognition:

1. What is the recognition rate of different symptoms of depression and schizophrenia in a sample of university students?

2. What are determining factors for recognition? Does faculty affiliation have an impact?

3. What conclusions can be drawn from these results for mental health policy?

## Methods

### Sampling and sample

This study is based on an online questionnaire completed by students of the University of Zurich/Switzerland. The baseline population included 2014 students in their 9^th ^or 10^th ^University semester (cut-off date: November 21^st^, 2003). The study sample was taken from a sub-population of 1228 students (61%) who had given permission that their e-mail address can be given out for research purposes. Students were then invited to participate by e-mail. Approval was granted by the University Legal Department. The anonymity of the participants was guaranteed.

The personal form of address was used. One of the authors (N.F.) introduced herself and gave preliminary information about the anonymity of the questionnaire. The test persons were assured they would receive feedback after the study terminated. Additionally, each session was introduced by questions from previous log-ins to control for repeated completion.

A pre-test with 34 students was preformed. Afterwards, the questionnaire was slightly modified with respect to the level of knowledge required and the wording of the questions. There was no criticism regarding the layout or the length of the survey. None of the test persons complained about the format of the questionnaire or the time it took to complete the study.

241 students completed the online-questionnaire (19.6% of the e-mail sub-population), three quarters within the first two days after receiving the invitation. 225 questionnaires were included (18.3% of the e-mail sub-population; 16 questionnaires were excluded due to missing data). The sample showed no statistically significant differences to the baseline population and the e-mail subpopulation regarding sex, age, faculty and main subject (Table 1). However, it cannot be excluded that knowledge differences exist between the two groups, for example their experience with mental disorders and the respective treatments.

**Table 1:** Demographic and study subject characteristics of the sample (N = 222–225)^1^

	Semester population	e-mail sample	Study sample
			
Sex	2012	1294	222
Women	1038 (51.6%)	651 (50.3%)	105 (52.7%)
Men	974 (48.4%)	643 (49.7%)	117 (47.3%)
			
Age	2012	1294	224
Mean (years)	27.14 (± 5.19)	27.01 (± 5.10)	26.41 (± 5.07)
			
Faculty	2012	1294	225
Law	377 (18.7%)	261 (20.6%)	37 (16.4%)
Economics	339 (16.8%)	254 (20.0%)	46 (20.4%)
Medical school	224 (11.1%)	137 (10.8%)	15 (6.7%)
Natural sciences^2^	182 (9.0%)	105 (8.3%)	18 (8.0%)
Philosophy / arts^3^	731 (36.3%)	429 (34.1%)	109 (36.0%)
Psychology	159 (7.9%)	108 (8.3%)	28 (12.4%)

^1 ^Varying sample sizes due to missing values

^2 ^6 students in veterinary medicine included

^3 ^1 student in theology included

### The questionnaire

The self-constructed questionnaire is comprised of the following parts:

1. Presentation of the survey aims and instructions for further proceedings.

2. Assessment of demographic factors (age, sex, faculty affiliation), personal experience with mental disorders and previous contact to people with mental illnesses (5 questions).

3. Assessment of knowledge about *depression *and *schizophrenia*:

• Prompting of 10 symptoms where 5 are part of the diagnostic criteria of the respective disease (according to the ICD-10 diagnostic criteria) and 5 are not (see Tables 2 and 3). Answer categories for each symptom according to the ICD-10 categorisation: main symptoms, additional symptoms and no symptoms of the respective disorder.

**Table 2:** Distribution of the interviewees who considered a symptom presented to be a main, an additional, or no symptom of *depression *(N = 221–225); only the first 5 symptoms are part of the diagnostic criteria of depression

	Main Sy.^1 ^(%)	Additional Sy.^2 ^(%)	False Sy.^3 ^(%)
	
Depressed mood	**209 (93.3%)**	13 (5.8%)	2 (0.9%)
Reduced energy	**200 (89.3%)**	22 (9.8%)	2 (0.9%)
Bleak and pessimistic views of the future	**192 (85.3%)**	33 (14.7%)	0 (0%)
Disturbed sleep	**112 (50.0%)**	101 (45.1%)	11 (4.9%)
Considerable distress or agitation	**84 (37.3%)**	125 (55.6%)	16 (7.1%)
			
Disorientation for the own person	1 (0.4%)	22 (9.8%)	**203 (89.7%)**
Compulsion to wash	0 (0%)	28 (12.3%)	**119 (57.6%)**
Disturbed perception	7 (3.1%)	62 (27.6%)	**158 (69.3%)**
Vague thinking and distorted speaking	7 (3.2%)	67 (30.3%)	**148 (66.5%)**
Repeated revival of a trauma	15 (6.8%)	138 (62.2%)	**69 (31.1%)**

^1 ^Main symptoms: respondents who considered the variables to be a main symptom of depression

^2 ^Additional symptoms: respondents who considered the variables to be an additional symptom of depression

^3 ^False symptoms: respondents who considered the variables not to be a symptom of depression

**Table 3:** Distribution of the interviewees who considered a symptom presented to be a main, an additional, or no symptom of *schizophrenia *(N = 222–225); only the first 5 symptoms are part of the diagnostic criteria of schizophrenia

	Main Sy.^1 ^(%)	Additional Sy.^2 ^(%)	False Sy.^3 ^(%)
	
Auditory hallucinations	**158 (70.5%)**	58 (25.9%)	8 (3.6%)
Feelings or actions experience as made or influenced by external agents^4^	**111 (49.6%)**	93 (41.5%)	20 (8.9%)
Delusions	**107 (47.8%)**	95 (42.4%)	22 (9.8%)
Delusions of control	**53 (23.8%)**	104 (46.6%)	66 (29.6%)
Hallucinations of taste	**42 (18.8%)**	110 (49.1%)	72 (32.1%)
			
Increased prevalence of allergies	2 (0.9%)	33 (14.7%)	**189 (84.4%)**
Agoraphobia with panic attacks	12 (5.3%)	74 (32.9%)	**139 (61.8%)**
Recklessly money spending in combination with grandiosity	21 (9.5%)	91 (41.0%)	**110 (49.5%)**
Both sex have an increased readiness for violence during and outside of illness episodes	17 (7.7%)	127 (57.2%)	**78 (35.1%)**
Split personality	144 (64.3%)	53 (23.7%)	**27 (12.1%)**

^1 ^Main symptoms: respondents who considered the variables to be a main symptom of schizophrenia

^2 ^Additional symptoms: respondents who considered the variables to be an additional symptom of schizophrenia

^3 ^False symptoms: respondents who considered the variables not to be a symptom of schizophrenia

^4 ^The so-called "Gefühl des Gemachten"

To avoid a bias due to the use of technical terms, we described the symptoms according to ICD-10. E.g., 'delusions' were explained as 'the feeling of being chased or threatened by an organisation like, e.g., the mafia without any external reason.' 'Depressed mood' was illustrated as 'feeling of continuous low mood that is rarely changing from day to day and unresponsive to and out of keeping with current circumstances.' However, to facilitate the understanding of the paper we consequently used the technical terms.

• One question each to assess the knowledge about lifetime prevalence (percentage of the general population), sex distribution (answers: more males, more females, no gender difference) and the age of the first episode of the respective disorder (age of onset). However, these data will be reported in a separate paper.

### Literacy scores

Three *literacy sum scores *were calculated. The first score, called true symptoms, was calculated by summing up each *correct positive assignment *of the items presented. Two points were given when an item correctly referred to the category main symptoms and one point for an item referring to the category additional symptoms. The maximum score that could be achieved was 10. The maximum score for false symptoms was five. The false symptoms score ('false symptoms') is representing the correct recognition of the items that are *not part of the diagnostic criteria *of the respective disorder (maximum score: 5). The overall score was calculated using the sum of 'true symptoms' and 'false symptoms'. Since the first and the second score had shown different results in bivariate and multivariate analysis the overall score was dropped from further analyses.

### Statistical analyses

The statistical analyses were carried out using SPSS for Macintosh (Version 6 and Version 11). The usual descriptive and bivariate analyses χ^2^-test, T-test and F-test were applied. Associations between faculty affiliation and depression/schizophrenia literacy were analysed by means of correspondence analysis [29]. Faculty affiliations were law, economics, medicine, natural and social sciences. Psychology was discriminated from social sciences. Literacy was analysed using a correspondence analysis of two 'interactive variables'. The first is matching gender and faculty affiliation and the second high/low literacy levels of depression and schizophrenia. The cut-off values with respect to the latter variable distinguished scores of ≤ 8 vs. >8 in depression (46.8% vs. 53.2% of the sample) and ≤ 6 vs. >6 in schizophrenia (51.4% vs. 48.6% of the sample) resulting in four categories (low/low, low/high, high/low and high/high literacy).

## Results

The recognition of depressive symptoms, whether main or additional, was consistent over 90% (Table 2). Depressed mood was well recognised as a symptom of depression whereas sleep problems, distress and agitation were only recognised as additional disorder. Repeated revival of a trauma was not well recognised as a symptom of depression. Schizophrenia compared to depression symptoms were not well recognised (Table 3). Split personality and increased readiness for violence were often mistaken for symptoms of schizophrenia.

Table 4 is shows the bivariate analyses of explanatory variables and the two knowledge scores for depression and schizophrenia symptoms, respectively. The mean sum scores are clearly higher for depression compared to schizophrenia indicating that symptoms of depression are better recognised. The probabilities of the T-tests and F-tests, respectively, indicate that true symptoms scores and false symptoms scores are related to different explanatory variables. This is despite the fact that all variables are more or less closely associated with the faculty affiliation. Medical and psychology students have the highest true symptoms scores. Their advantage is more noticeable with respect to schizophrenia than to depression. However, medical students do not differ from other faculties regarding false symptoms scores.

**Table 4:** Bivariate analyses (t-test or F-test) of explanatory variables and knowledge mean sum scores regarding depression and schizophrenia (N = 222–225)

		Sample	Depression		Schizophrenia	
				
		N^1 ^(%)	True symptoms^2^	False symptoms^3^	True symptoms^2^	False symptoms^3^
				
Sex	Men	105 (47.3%)	8.2	3.4	5.9	2.6
	Women	117 (52.7%)	8.6	3.4	6.5	2.3
	p		**.007**	**.903**	**.059**	**.122**
				
Age-group	-24	147 (65.9%)	8.4	3.4	6.1	2.3
	25–29	57 (25.6%)	8.5	3.5	6.5	2.6
	30+	19 (8.5%)	8.4	3.9	7.0	2.7
	p		**.917**	**.183**	**.163**	**.312**
				
Faculty	Law	37 (16.4%)	8.7	3.3	6.0	2.5
	Economics	46 (20.4%)	8.2	3.2	5.7	2.0
	Medical school	15 (6.7%)	9.0	3.1	8.2	1.7
	Natural sciences^4^	18 (8.0%)	8.3	3.3	6.0	2.4
	Philosophy / arts^5^	109 (48.4%)	8.3	3.4	5.7	2.4
	Psychology	28 (12.4%)	8.6	4.5	8.4	3.4
	p		**.081**	**.000**	**.000**	**.000**
				
Previous interest in this subject	yes	105 (46.7%)	8.6	3.6	7.2	2.6
	no	122 (53.3%)	8.3	3.4	5.5	2.3
	p		**.026**	**.221**	**.000**	**.029**
				
Side job related to mental disorders	yes	33 (14.7%)	9.0	3.5	8.1	2.7
	no	192 (84.6%)	8.3	3.4	5.9	2.4
	p		**.003**	**.631**	**.000**	**.198**
				
Self-experience of mental illness	yes	133 (58.6%)	8.5	3.6	6.5	2.4
	no	92 (40.5%)	8.3	3.3	5.9	2.5
	p		**.386**	**.078**	**.070**	**.675**
				
Own treatment experience due to mental problems	yes	44 (19.4%)	8.6	3.8	7.3	2.7
	no	192 (79.7%)	8.4	3.4	6.0	2.4
	p		**.254**	**.042**	**.001**	.**069**
				
Knowledge about mental disorders	yes	122 (53.7%)	8.4	3.7	6.8	2.6
	no	103 (45.4%)	8.4	3.1	5.7	2.2
	p		**.957**	**.000**	**.001**	**.037**

^1 ^Varying sample sizes due to missing values

^2 ^True symptoms: mean sum score about the recognition of the items that are part of the diagnostic criteria of depression or schizophrenia, respectively

^3 ^False symptoms: mean sum score about the recognition of the items that are not part of the diagnostic criteria of depression or schizophrenia, respectively. A high false negative symptoms score reflects greater mental health literacy.

^4 ^6 students in veterinary medicine included

^5 ^1 student in theology included

The mean true symptoms score for depression was 8.42 (± 1.17) and for schizophrenia 6.27 (± 2.32), respectively. The mean false symptoms score for depression was 3.45 (± 1.24) and for schizophrenia 2.43 (± 1.15), respectively. In both depression and schizophrenia symptoms score, the correlations between the positive and negative symptoms scores were not significantly different from 0.

### Correspondence analysis

The correspondence analysis of the interactive variables gender*faculty (e.g., m_law) vs. depression*schizophrenia (e.g., LOWLOW) literacy yielded a total inertia of 0.319 (inertia represents the percent of variance explained by each dimension). The inertia of the first dimension was 0.203 (64% of total inertia) and of the second 0.098 (31%). The contribution of the distinct categories to the inertia of each dimension is depicted in Table 5.

**Table 5:** Contribution of each dimension to the inertia*

		Dimension	
Variable		1	2
		
depression*schizophrenia literacy	11 LOWLOW	.360	.358
	12 LOWHIGH	.053	.055
	21 HIGHLOW	.104	.520
	22 HIGHHIGH	.483	.067
			
gender*faculty affiliation	12 m_law	.000	.246
	13 m_econ	.181	.143
	14 m_med	.114	.035
	16 m_scie	.027	.113
	17 m_arts	.092	.037
	18 m_psyc	.083	.025
	22 f_law	.042	.089
	23 f_econ	.008	.012
	24 f_med	.220	.036
	26 f_scie	.002	.181
	27 f_arts	.041	.049
	28 f_psyc	.190	.034

* Inertia: represents the percent of variance explained by each dimension

Figure 1 shows how gender and faculty diverge among the categories of literacy. There are three different groups. Medical and psychology students had an overall high level of literacy. Male students of natural sciences, economics and philosophy were especially illiterate.

## Discussion

Mental health literacy has been recognised as a crucial prerequisite for early recognition and intervention in mental disorders. In this online-based questionnaire we presented symptoms of depression and schizophrenia to a sample of university students. The vast majority of the participants recognised the specific symptoms of depression. The symptoms of schizophrenia, however, were recognised to a lesser extent. Delusions of control and hallucinations of taste were not identified as symptoms of schizophrenia. In contrast, repeated revival of a trauma for depression and split personality for schizophrenia were mistaken as symptoms of the respective disorder. Bivariate analyses demonstrated that faculty affiliation, not gender, age or personal experience of mental illness, had an influence on symptom recognition in either disorders. Previous interest in and a side job related to mental disorders as well as treatment experience had a positive influence on symptom recognition. The correspondence analysis showed that male students in natural sciences, economics and philosophy are especially illiterate in recognising the two disorders. Gender became an important factor when controlling for faculty affiliation.

Thus, our questions can be answered as follows: the recognition rate of diverse symptoms of depression and schizophrenia among university students varies according to gender, symptoms and the faculty affiliation. It is also dependent on previous experience with mental disorders, be it at work, by interest or due to own treatment experience. Gender and faculty affiliation play a deciding role in recognising mental disorders.

### Methodological considerations

As far as it is known, this is the first online survey in the field of mental health literacy. The Internet is likely to facilitate access to people and information. Thus, interest in online surveys are growing despite some shortcomings, e.g., sampling limitations as a result of omitting those without an email address/access as well as limits on response alternatives and interviewer observation [30]. Interpretation of responses is difficult because we do not know whether the answers were honest, i.e. whether the interviewees responded according to their own knowledge or if they made use of additional information about mental disorders – although they were instructed not to do so. There are limitations concerning public opinion surveys: they may be criticised for underestimating antipathy as those unaffected or disinterested may refuse to participate in a survey and many give socially desirable responses [31]; medical researchers tend to ask closed questions and obtain positive answers while sociologists ask open question, thus, uncovering negative stigmatising answers [32]; and attitudes should not be mistaken for actual interpersonal behaviour, but should be considered as a 'proxy' measure of social behaviour [33]. Furthermore, one could wonder whether the response rate is not higher than 18.3% of the eligible sample. However, our response rate is higher than previous online surveys at the University of Zurich (Zurich University administration, personal communication, 2003) and it must be mentioned that no incentives for participating were given. The schizophrenia symptoms did not include negative symptoms. We believe that the distinction between negative symptoms of a patient with schizophrenia and some symptoms of depression are too specific to be asked in an online survey among lay people, and could be a source of bias.

### Comparison to the literature

There are few publications about how the general population recognises mental disorders. Four in ten adults who are symptomatic, but undiagnosed, have never heard of clinical depression and 84% had never heard of anxiety disorders [34]. In an Australian survey, nearly 38% of those questioned did not recognise depression [35]. Magliano et al. [36] found in a survey conducted in Italy that 21% of the general public identified schizophrenia in the case vignette. In a comparable opinion poll in Switzerland, the depression vignette was correctly recognised by 39.8% of the respondents whereas the remaining 60.2% considered the person depicted as having a 'life crisis' [37]. Schizophrenia, however, was recognised by 73.6% of the interviewees. Recognition of mental disorders was closely related to positive attitudes of psychiatry in general, but not to previous experience with treatment [38].

Our results show low recognition of distinct symptoms of mental disorders, which differs from the findings when a vignette was presented. In the latter, symptoms of schizophrenia were recognised as an illness and specific symptoms were recognised at a low rate. The symptoms of depression showed exactly the opposite results: Specific symptoms were recognised as illness-related whereas in the vignette the symptoms were classified as a 'life crisis'. For mental health policy recommendations and awareness campaigns to be useful special emphasis must be given on the *differences between depression and schizophrenia*.

### Implications for mental health policy

Based on both the literature and the results presented in this study recommendations for mental health policy can be assessed. Correspondence analysis yields two substantial effects: education and gender-related effects. The major effect of education is very important, for example the study of medicine or psychology. But one must not forget that to study on the university is a privilege and, therefore, the tested student population is not representative of the population in general. The differences in mental health literacy would probably be even more obvious. Thus, one conclusion is that *education *can be very important when changing prejudice [39].

When comparing male and female students regardless of faculty, except medicine and psychology, females had a higher mental health literacy. This *gender-specific effect *is in line with research findings of gender differences concerning knowledge about and attitudes towards people with mental illness. Women tend to react more sympathetic towards people with mental illness and are more likely to volunteer in psychiatry wards or hospitals [40]. Therefore, gender-specific interventions should be considered.

What should be part of these lessons? The knowledge of the symptoms of *depression *is considerably high which could be a result of the high prevalence of affective disorders [41]. This seemingly contrasts with the findings that depression is hardly considered as a mental illness [42]. In general, depressive states are not viewed as illnesses but rather as normal psychological phenomena commonly called life crises [43]. This could also explain why a 'repeated revival of a trauma' is often mistaken as a symptom of depression. The consequences of public unawareness is that medical treatment for depression is not regarded as being necessary while non-medical interventions are thought to be helpful [44]. Thus, for mental health policy the emphasis on *categorisation of depression as a mental illness *(and not as a 'life crisis') and to a lower extent the recognition of distinct symptoms is very important.

Further suggestions for improving health literacy concerning *schizophrenia *are based on different findings: the *low recognition *rate of symptoms of schizophrenia compared with depression and two stereotypical attitudes about schizophrenia, namely, that it is associated with more violence and that split personality is one of its main symptoms. Firstly, the recognition is influenced by interest and job experience. Thus, previous practical or theoretical *contacts *to the topic improve the recognition of the disorder and its respective symptoms. This supports the findings that in the general population contact with mentally ill people is increasing along with the recognition of their disorder [45]. Additionally, contact has been shown to positively influence various parameters concerning people with mental illness, e.g., social distance or restrictions towards the mentally ill [39,46-48].

*Stereotypes *such as 'people with schizophrenia areviolent' or 'have a split personality' must be addressed. Whenever people with schizophrenia are more violent than the general public, it involves a combination of factors, for example male gender, comorbid substance use, less treatment compliance or severe psychopathology [49]. These constantly disseminated stereotypes can only be fought by persistent and differentiated *information and clarification*. This proposal is in line with previous findings that information improves mental health literacy [39]. In this regard, the mass media may play a crucial role. Instead of demonising people with mental illnesses and, thus, increasing prejudices against the affected, they should be motivated to engage in the fight against discrimination and stigmatisation of mental illness.

## Conclusion

There is a lack of knowledge among university students, especially among those in natural sciences, philosophy and economics. Differences between distinct groups among the general population are expected to be even more extreme. Poor mental health literacy was significant regarding symptoms of schizophrenia. The following conclusions can be drawn to improve mental health literacy:

• Contact to mental disorders, either in a theoretical way (e.g., by interest) or by practical activity (e.g., at work), improves mental health literacy. Thus, possibilities for contact must be available.

• As it is true for health in general [50], education regarding mental health is needed. The time before university is advantageous, either in high school or during the apprenticeship.

• Education should emphasise that depression is a mental illness

• Stereotypes of schizophrenia should be addressed.

## Competing interests

The author(s) declare that they have no competing interests.

## Authors' contributions

C.L., V.A.-G. and W.R. designed the study and drafted the paper. N.F. collected the data and gave significant contributions to the content of the paper. N.S. gave significant contributions to the content of the paper and did the final editorial revision. All authors read and approved the final manuscript.

## Pre-publication history

The pre-publication history for this paper can be accessed here:


